# *Ex Vivo* Modeling of Chemical Synergy in Prenatal Kidney Cystogenesis

**DOI:** 10.1371/journal.pone.0057797

**Published:** 2013-03-12

**Authors:** Corina Anders, Nick Ashton, Parisa Ranjzad, Mark R. Dilworth, Adrian S. Woolf

**Affiliations:** 1 Institute of Human Development, Faculty of Medical and Human Sciences, University of Manchester, Manchester Academic Health Science Centre and St Mary's and Manchester Children's Hospital, Manchester, United Kingdom; 2 Faculty of Life Sciences, University of Manchester, Manchester, United Kingdom; Mario Negri Institute for Pharmacological Research and Azienda Ospedaliera Ospedali Riuniti di Bergamo, Italy

## Abstract

Cyclic adenosine monophosphate (cAMP) drives genetic polycystic kidney disease (PKD) cystogenesis. Yet within certain PKD families, striking differences in disease severity exist between affected individuals, and genomic and/or environmental modifying factors have been evoked to explain these observations. We hypothesized that PKD cystogenesis is accentuated by an aberrant fetal milieu, specifically by glucocorticoids. The extent and nature of cystogenesis was assessed in explanted wild-type mouse embryonic metanephroi, using 8-Br-cAMP as a chemical to mimic genetic PKD and the glucocorticoid dexamethasone as the environmental modulator. Cysts and glomeruli were quantified by an observer blinded to culture conditions, and tubules were phenotyped using specific markers. Dexamethasone or 8-Br-cAMP applied on their own produced cysts predominantly arising in proximal tubules and descending limbs of loops of Henle. When applied together, however, dexamethasone over a wide concentration range synergized with 8-Br-cAMP to generate a more severe, glomerulocystic, phenotype; we note that prominent glomerular cysts have been reported in autosomal dominant PKD fetal kidneys. Our data support the idea that an adverse antenatal environment exacerbates renal cystogenesis.

## Introduction

Polycystic kidney diseases (PKDs) cause major morbidity and mortality. For example, around 6% of adults starting long-term renal replacement therapy have PKD [1]. Most such cases are autosomal dominant PKD (ADPKD) whereas in children autosomal recessive PKD (ARPKD) more often causes end-stage renal disease than ADPKD [1]. ADPKD is caused by mutations of *Polycystic Kidney Disease 1* (*PKD1*) or *PKD2* [2], and ARPKD is caused by *Polycystic Kidney and Hepatic Disease Gene 1* (*PKHD1*) mutations [3]. Current thinking is dominated by the concept that renal cyst formation and growth in PKD is determined by perturbed epithelial biology resulting from these mutations [2], [4]. Indeed, all three genes are normally expressed in kidney tubules and encode proteins located in primary cilia, organelles thought to act as mechano- and/or chemo-sensitive transducers regulating epithelial cell turnover and differentiation [4].

Individuals with ARPKD who are born with massive nephromegaly generally have non-functional *PKHD1* mutations, while missense mutations are associated with milder kidney disease [5]. Different *PKHD1* mutations cannot, however, explain the spectrum of severity of renal cystogenesis documented to occur within certain ARPKD families [6], [7]. Similarly, the severity of cystic kidney disease can vary markedly between affected individuals within a single ADPKD kindred (8). In some cases, the severe kidney phenotypes in such families have been demonstrated to occur in individuals who also harbor a pathogenic variant of a modifying gene such as *Hepatocyte Nuclear Factor 1B* (*HNF1B*) [9], which codes for a transcription factor regulating *PKD* and other epithelial genes [10], [11].

It has also been suggested [8] that yet-to-be defined non-genomic factors also contribute to variations in the severity of nephropathy within PKD families. Our hypothesis is that the fetal milieu influences the tempo of kidney cystogenesis. When developing kidneys of wild-type animals are exposed to adverse environments, their growth and differentiation trajectories deviate from normal [12], [13]. Low protein diet (LPD) administered to pregnant animals is a much studied model of fetal programming [14]. Maternal LPD alters gene expression and precursor cell turnover in the offspring's embryonic kidneys [15], [16]; it also perturbs physiological functions in mature kidney tubules [17], [18]. LPD reduces placental expression of 11β hydroxysteroid dehydrogenase type 2 (11β-HSD2) [19], an enzyme which degrades and protects embryos from overexposure to maternal glucocorticoids. Notably, exposure of explanted wild-type metanephroi to high glucocorticoid concentrations is cystogenic [20], [21].

Numerous studies support the conclusion that growth of renal cysts in various genetic forms of PKD is, at least in part, driven by cyclic adenosine monophosphate (cAMP) acting within tubule epithelia [22]–[24]. Furthermore, cAMP analogues are cystogenic in wild-type metanephric organ culture [25]. Here, we demonstrate, for the first time, that glucocorticoids strikingly synergize with cAMP to cause cyst growth in wild-type embryonic kidneys, an observation which supports the idea that an adverse antenatal environment can exacerbate genetic cystic disease.

## Materials and Methods

Ethics Statement: Animal studies using Schedule-1 killing of wild-type CD1 mice maintained in our local Biological Services Colony were ethically approved by the Registered Medical and Scientific Departments of The University of Manchester, UK. Reagents were obtained from Sigma Chemical (Poole, UK), unless stated. The morning after mating was designated as E0, and freshly isolated metanephroi (E13) were explanted on Millicell inserts (Millipore, Bedford, MA) and grown at 37°C in a humidified atmosphere of air-5% CO_2_. Explants were fed with defined, serum-free medium comprising DMEM/F12 (GIBCO BRL, Gaithersburg, MD), insulin (10 mg/l), sodium selenite (5 µg/l), and transferrin (5.5 mg/l) [21]. In some experiments, this vehicle/control medium was supplemented with one or more of the following chemicals: dexamethasone (4.7 or 47 or 470 nM), 8-Br-cAMP (1–100 µM), [Arg8] vasopressin (0.1 or 1 µM) [26], forskolin (10 µM), Z-VAD-FMK (25 µM or 100 µM; R&D systems FMK001), a general caspase inhibitor [27]; sorafenib tosylate (100 nM or 100 µM; Nexavar, Bayer 43-9006) an inhibitor of the Raf/Mek/Erk pathway (MAP kinase) pathway [28]. All media components were changed after three days. Metanephroi were photographed as whole-mounts at day 0, 3 and on the final day of the 6-day culture period and the number of cyst-like structures were counted, and the organ size measured, using ImageJ [21] and Adobe Photoshop software. These measurements were undertaken by an operator unaware of the culture conditions to which each organ had been exposed. Data were analyzed using the Kruskal-Wallis test followed by Mann-Whitney tests to identify individual differences, with the Holm's sequential Bonferroni correction to control for the family wise (false-discovery) error rate.

Paraformaldehyde-fixed kidneys were embedded in paraffin and sectioned at 4 µm and, after dewaxing, were counterstained with hematoxylin. In some sections, immunohistochemistry was performed for: aquaporin-1 (Abcam ab15080), calbindin-28 (Abcam ab25085), megalin (Acris Antibodies DM3613P), phospho-histone H3 (Abcam ab5176), uromodulin (Santa Cruz sc-20631), the macrophage marker F4/80 (Abcam ab6640) and the M2 macrophage marker, the mannose receptor/CD206 (Abcam ab8918 and Abdserotec MCA2235GA). Primary antibodies were detected using appropriate secondary antibodies and a peroxidase-based system, generating a brown colour [21]. Negative controls consisted of omission of primary antibodies and these experiments showed no significant signal (data not shown). Other sections were probed with *Dolichos biflorus* agglutinin (Vector Laboratories B-1035) and this was detected with a peroxidase-based system. To detect apoptotic nuclei, the Fluorescein In Situ Cell Death Detection Kit (Roche) was used. Total glomerular numbers in organs cultured for three days were measured by counting glomeruli in every second 4 µm section pair using a modified version of the physical fractionator technique [29]. The number of glomeruli per organ was calculated using the formula described by Dilworth et al [30].

## Results

8-Bromoadenosine 3′, 5′-cAMP (8-Br-cAMP) is a membrane-permeable cAMP analogue. When added to media at a concentration of 100 µM, it is cystogenic in wild-type mouse metanephroi explanted and maintained in organ culture [25]. Moreover, exposure of cultured wild-type metanephroi to 470 nM dexamethasone, a synthetic glucocorticoid, is itself cystogenic [21]. Here, we quantified and compared effects of each agent on embryonic day 13 wild-type mouse metanephric kidneys which were fed serum-free defined media for up to six days. Furthermore, the possible effects of lower concentrations (4.7 or 47 nM) of dexamethasone were examined. As explained in the *Discussion*, this range of dexamethasone concentrations encompasses the range of glucocorticoid activity found in the human circulation. To determine the effects of co-administration of cAMP and glucocorticoids, metanephric explants were exposed to 100 µM 8-Br-cAMP supplemented with 4.7, 47 or 470 nM of dexamethasone.

**Figure 1:**
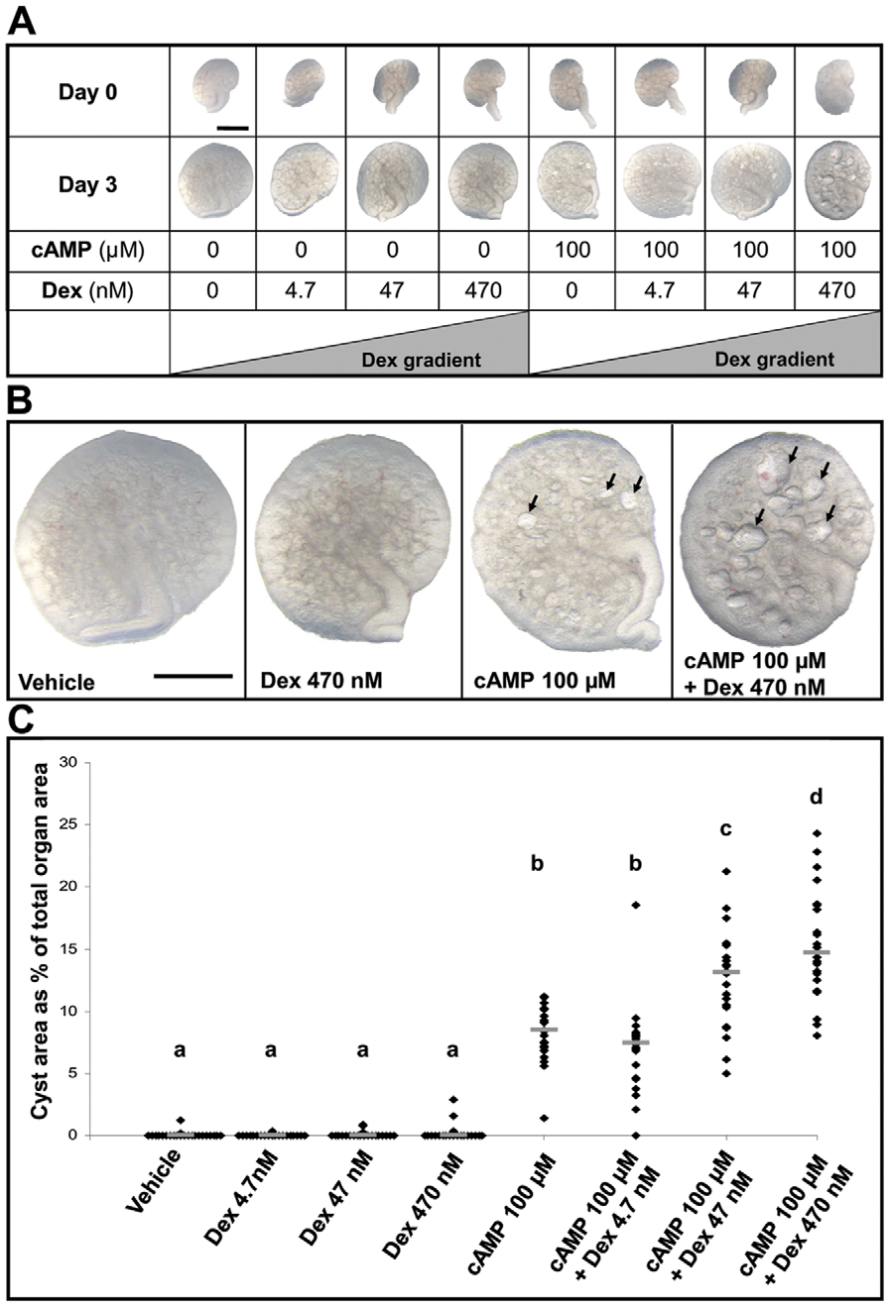
Whole mount of E13 metanephroi cultured for three days. **A.** Experimental schema showing the eight different conditions studied with, for each, whole mount images of a representative organ at Day 0 and the same organ at Day 3. Bar is 500 µm. **B.** Higher power whole-mount views of representative organs from control (*Vehicle*), 470 nM dexamethasone (*Dex*) alone, 100 µM 8-Br-cAMP (*cAMP*)-only, or co-treatment with both. In this illumination, cystic structures appear as pale circles or ovals, and some of these are arrowed. Bar is 500 µm. **C.** Percentage of total explant area occupied by cystic structures. Each ♦ represents the value for a separate organ (n = 21 to 23 for each condition), with bars indicating group medians. Above each group is a letter; those designated by the same letter (e.g. “a”) are not significantly different from each other. In contrast, groups designated by different letters are significantly (P<0.05) different from each other (e.g. those marked “a” are different from all the other groups designated “b”, “c” and “d”).

**Figure 2:**
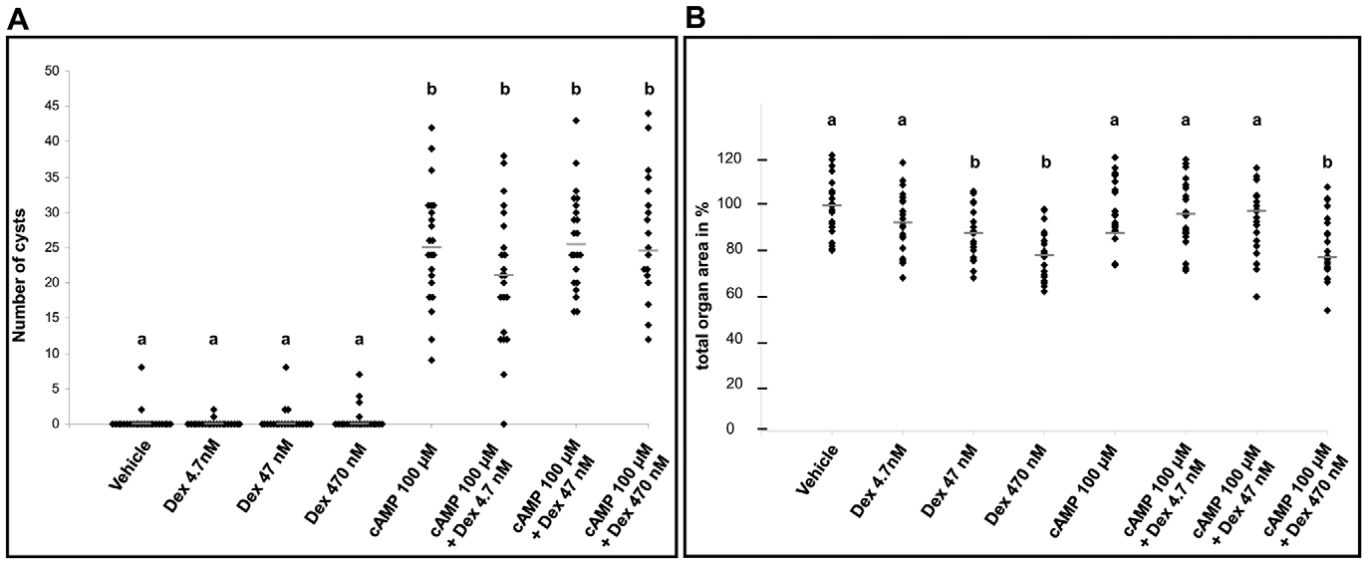
Cyst numbers and organ areas on day three of culture. **A.** Total numbers of cysts at day three of culture. **B.** Areas of organs at day three of culture expressed relative to those exposed to vehicle-only. For both A. and B., each ♦ represents the value for a separate organ (n = 21 to 23 organs for each condition), with bars indicating group medians. Groups designated by the same letter (e.g. all those designated by “a”) are not significantly different from each other. In contrast, groups designated by different letters are significantly different (P<0.05).

**Figure 3:**
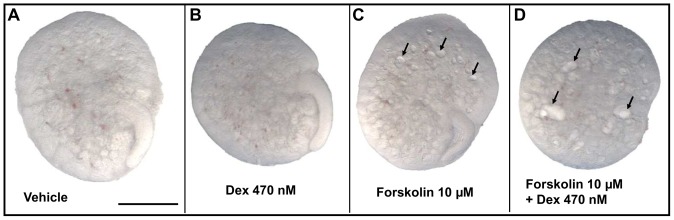
Effects of forskolin on day three of culture. Higher power views of typical organs from vehicle (**A**), 470 nM dexamethasone alone (**B**), 10 µM forskolin-only (**C**), or co-treatment with both (**D**). Cystic structures appear as pale circles or ovals, and some of these are arrowed. Bar is 500 µm.

On day three of culture, as assessed by direct observations of living explants (Figure 1A–C and Figure 2), dexamethasone alone was not cystogenic at any concentration. The two highest glucocorticoid concentrations (47 or 470 nM) did, however, result in small but significant reductions in explant areas versus exposure to vehicle-alone or 4.7 nM dexamethasone. Exposure to 100 µM 8-Br-cAMP alone was cystogenic, generating a median of 25 cyst-like structures per organ; these cysts collectively occupied a median of 8% of the area of each kidney. When either 4.7, 47 or 470 nM dexamethasone was added to 100 µM 8-Br-cAMP, the numbers of cyst-like structures per organ (respective medians of 21, 25 and 24) were not significantly different compared with 100 µM 8-Br-cAMP alone. In combination with 100 µM 8-Br-cAMP, however, each of the two higher dexamethasone concentrations resulted in significant concentration-dependent increases in the areas of organs occupied by cystic structures (the medians for 47 nM and 470 nM were respectively 13% and 15%). In the latter condition, there was a modest but significant reduction in explants size compared with 100 µM 8-Br-cAMP alone or this chemical supplemented with either 4.7 or 47 nM dexamethasone. Qualitatively similar observations with regard to cystogenesis were made when 100 µM 8-Br-cAMP was substituted with 10 µM forskolin, a chemical which raises intracellular cAMP levels. Exposure to forskolin alone generated cysts (Figure 3) and these effects appeared more prominent when it was applied together with 470 nM dexamethasone. Exposure to [Arg8] vasopressin, an AVP V_2_ receptor agonist, applied at 0.1 or 1 µM [26], failed to result in formation of cystic structures (data not shown).

**Figure 4:**
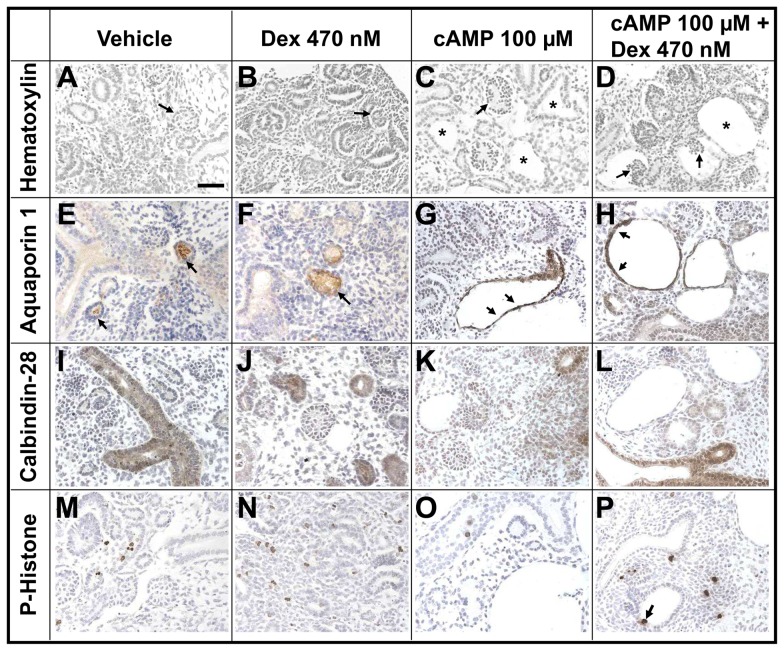
Histology of explants cultured for three days. Representative images are shown for 3–5 organs which have been examined in each condition. All sections counterstained with hematoxylin (blue nuclei). **A–D.** Note lack of cysts in control (*Vehicle*) and 470 nM dexamethasone (*Dex*)-only exposed organs, with plentiful dilated tubules and cysts (some of which are indicated by asterisks) in rudiments exposed to 100 µM 8-Br-cAMP (*cAMP*)-only, or co-treated with both chemicals. In these frames arrows point to glomeruli; note the “glomerulocystic” phenotype in explants exposed to both 8-Br-cAMP and glucocorticoid. **E–H.** Tubules and cysts reactive (brown) with antibody to aquaporin-1 are indicted by arrows. **I–L.** Brown colour indicates tubules immunoreactive with calbinin-28 antibody. Note that cyst epithelia are negative. Calbinin-28 was prominently detected in non-dilated tubules in all four conditions. **M–P.** Nuclei that are brown have reacted with phospho-histone antibody. Note the prominent proliferation within the stromal compartment in organs exposed to 470 nM dexamethasone, either alone (N) or with 8-Br-cAMP (P). In P, the arrow indicates a rare labeled nucleus in cyst epithelia. Bar is 50 µm.

**Figure 5:**
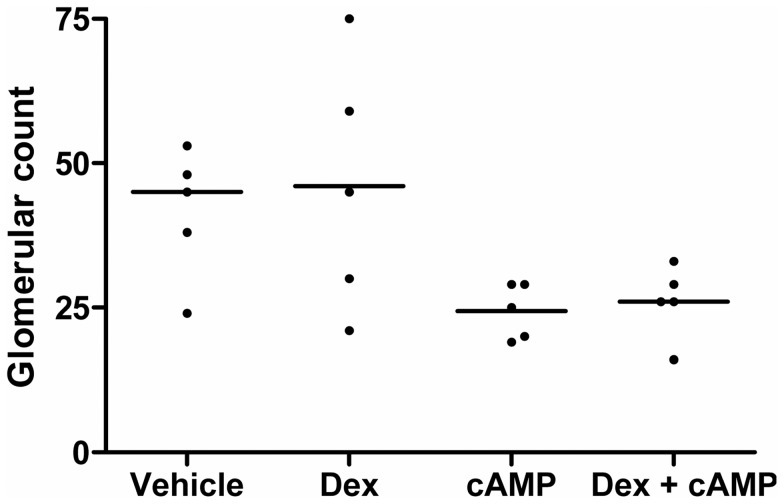
Glomerular numbers in explants. Each point represents the number of glomeruli in a metanephros after three days of culture, as measured using a modified version of the physical fractionator technique. Bars indicate the median values of the experimental groups (n = 5 organs for each condition). Although there tended to be fewer glomeruli in the 8-Br-cAMP-exposed groups, the culture conditions had no significant effect on glomerular numbers (Kruskal-Wallis test P = 0.065). Key: *Dex* = 470 nM dexamethasone and *cAMP* = 100 µM 8-Br-cAMP.

Day three explants were then analyzed using histology (Figure 4). At the start of the culture period, each wildtype mouse E13 metanephros contains a ureteric tree which has branched several times, together with renal mesenchyme which has begun to differentiate into nephron precursors (so-called vesicles, comma- and S-shaped bodies); glomeruli, however, have yet to form [21]. After three days of culture, vehicle-only exposed rudiments contained glomeruli and tubules separated by stromal cells (Figure 4A). Grossly similar histological features were noted in dexamethasone-only exposed rudiments (Figure 4B). Explants exposed to 100 µM 8-Br-cAMP, either alone or in combination with dexamethasone, contained dilated tubules and cysts (Figure 4C and D). Strikingly, in cultures co-treated with 100 µM 8-Br-cAMP and 470 nM dexamethasone, some cyst profiles were seen to contain a glomerular tuft attached to the cyst wall (Figure 4D); this “glomerulocystic” phenotype was not detected in cysts arising in explants exposed to 100 µM 8-Br-cAMP alone. As measured using a modified version of the physical fractionator technique [29], [30], there tended to be fewer glomeruli in the 8-Br-cAMP-exposed groups (Figure 5); the medians (ranges) were: vehicle-only, 45 (24–53); dexamethasone alone 45 (21–75); 8-Br-cAMP alone, 25 (19–29); and 8-Br-cAMP plus dexamethasone, 26 (16–33). However, the culture conditions had no significant (Kruskal-Wallis test P = 0.065) effect on glomerular numbers.

**Figure 6:**
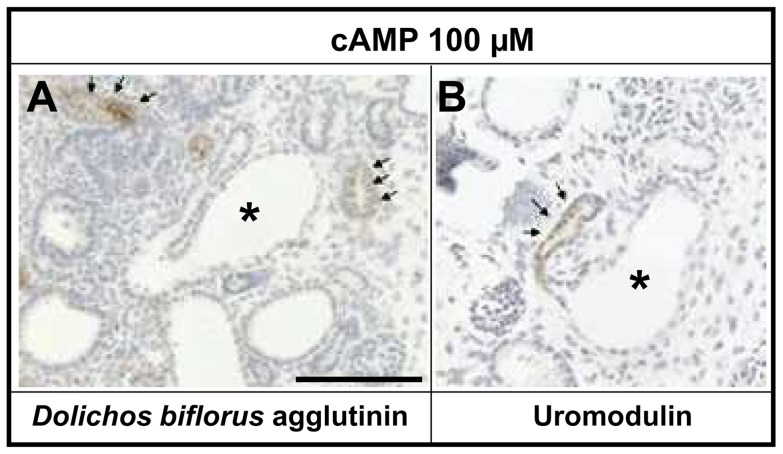
Histology of day three rudiments exposed to 100 µM 8-Br-cAMP (*cAMP*)-only. **A and B.** Adjacent sections counterstained with hematoxylin. In A, several non-dilated tubules are stained brown (arrows), having bound *Dolichos biflorus* agglutinin; they most likely represent collecting ducts. The centre of each frame is dominated by a tubule which has a “U-turn”. Uromodulin was immunolocalised (brown color) in the undilated limb (indicated by the arrows in B). In contrast, epithelia in the adjacent limb, which is dilated (asterisk), were unreactive with the uromodulin antibody. The simplest deduction is that the dilatation is present in the descending limb of the loop of Henle. Bar is 100 µm.

Sections of vehicle-only, 470 nM dexamethasone-only, 100 µM 8-Br-cAMP-only and 8-Br-cAMP plus dexamethasone day three cultures were probed with antibodies to: megalin, to define proximal tubules [31]; aquaporin-1, to define proximal tubules and thin descending limbs of loops of Henle [32], uromodulin, to detect thick ascending limbs of loops of Henle [33]; and calbindin-28, which is reported to be located in both the distal part of the developing nephron and also the collecting duct in mouse metanephroi [34]. Other sections were reacted with *Dolichos biflorus* agglutinin which binds collecting duct stalks [35]. In explants exposed to only vehicle or dexamethasone, a subset of tubules expressed aquaporin-1 and megalin, as did most cysts in explants treated with 100 µM 8-Br-cAMP alone or this nucleotide plus 470 nM dexamethasone (Figure 4E–H). In contrast, at this time-point, no cysts labeled with calbindin-28 (Figure 4I–L) or *Dolichos biflorus* agglutinin (Figure 6A), although each probe labeled non-dilated tubules in explants in all four conditions. In occasional histology sections of cystic kidneys, a subset of tubules below the nephrogenic zone were observed to have a sharp bend, reminiscent of the U-bend in loop of Henle. Some of the loops contained (only) one limb which was dilated; we deduced that this must be the descending limb because uromodulin was immunodetected in the opposite, non-dilated limb (Figure 6B). Proliferating cells, as assessed by phospho-histone expression (Figure 4M–P), were detected in epithelia and stroma in control explants. Proliferation appeared prominent within the stroma of metanephroi exposed to dexamethasone and was noted in a small subset of cyst epithelial cells. Exposure of explants to Sorafenib Tosylate (100 nM or 100 µM), an inhibitor of the Raf/Mek/Erk (MAP kinase) pathway [28], resulted in severe inhibition and distortion of organogenesis, rendering it impossible to assess any specific effects on cystogenesis (data not shown).

**Figure 7:**
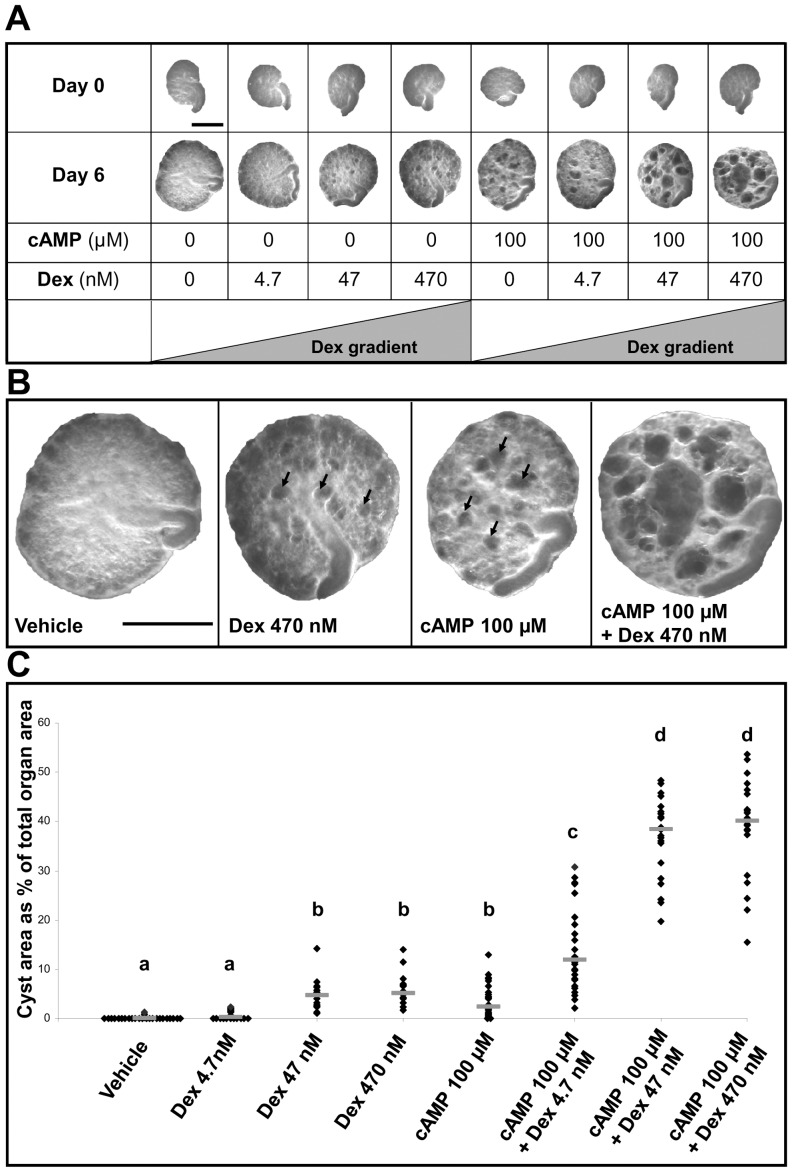
Whole mounts of E13 metanephroi cultured for six days. **A.** A representative organ at Day 0 and the same organ at Day 6 for each condition. Bar is 500 µm. **B.** Enlarged images of a typical organ from control (*Vehicle*), 470 nM dexamethasone (*Dex*) alone, 100 µM 8-Br-cAMP (*cAMP*)-only, or co-treatment with both. Cysts appear in this dark field illumination as black circles or ovals. Bar is 500 µm. **C.** Percentages of total explant areas occupied by cysts. Each ♦ represents the value for a separate organ (n = 21 to 28 organs for each condition), with the bars indicating the group medians. Above each group is a letter; those designated by the same letter (e.g. “a”) are not significantly different from each other. In contrast, groups designated by different letters are significantly (P<0.05) different from each other (e.g. those marked “a” are different from all the other groups designated “b”, “c” and “d”).

**Figure 8:**
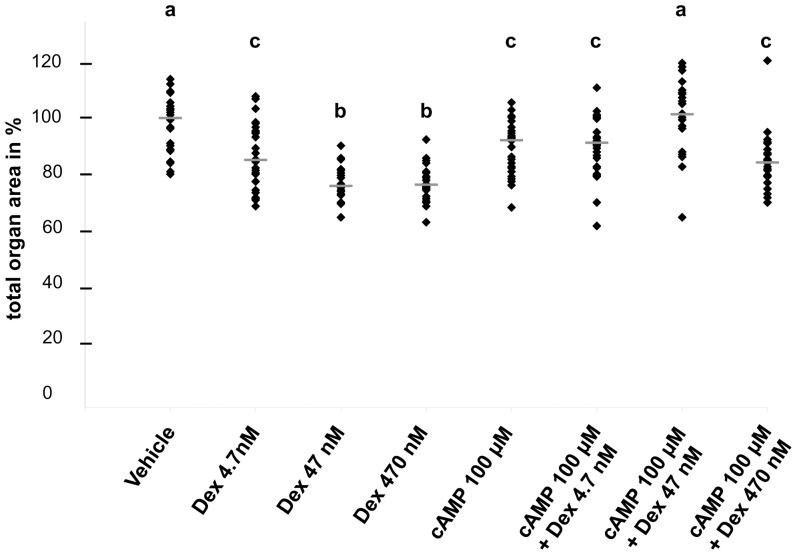
Organ areas at day six of culture. Areas of explants at day six expressed relative to those exposed to vehicle-only; key as for 2B.

**Figure 9:**
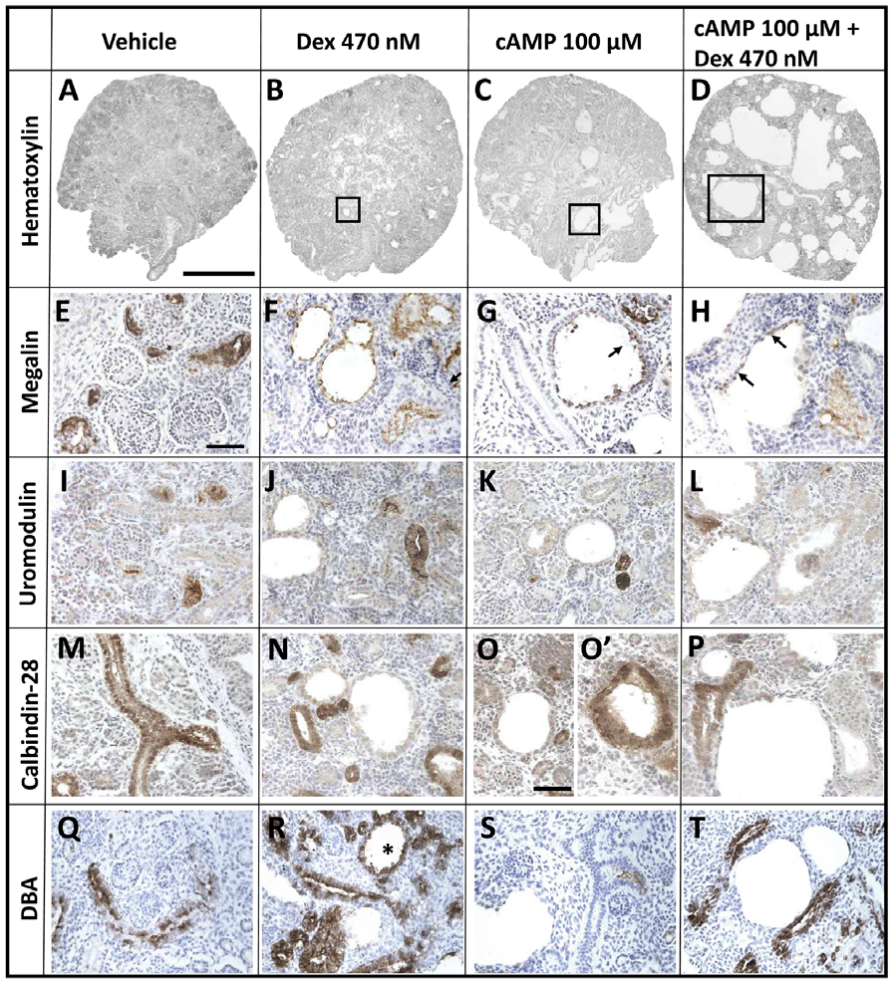
Histology of explants cultured for six days. Representative images for 3–5 organs in each condition. All sections counterstained with hematoxylin (blue nuclei). **A–D.** Note lack of cysts in control (*Vehicle*) explants and the progressively greater size (a typical cyst is boxed in each image) and extent of cysts per organs exposed to 470 nM dexamethasone (*Dex*)-alone, 100 µM 8-Br-cAMP (*cAMP*)-only, or both chemicals. **E–H.** Brown color indicates immunoreactivity to megalin antibody. Note patchy reactivity of cyst epithelia in organs exposed to 470 nM dexamethasone-alone, 100 µM 8-Br-cAMP-alone or both chemicals. **I–L.** Uromodulin immunoreactivity (brown); note that cysts are not labeled. **M–P.** Immunostaining with antibody to calbindin-28. Most cyst epithelia are unreactive (M, O and P) but a small subset of cysts in cAMP-exposed organs were positive and one such is depicted in O′. **Q–T.** These sections were probed with *Dolichos biflorus* lectin. Note that the lectin prominently labeled non-dilated tubules between cysts; in addition, the great majority of cysts did not bind the lectin. In (R), an asterisk indicates a rare, labeled cyst. Bar in A–D is 500 µm, and bar for other frames is 50 µm.

**Figure 10:**
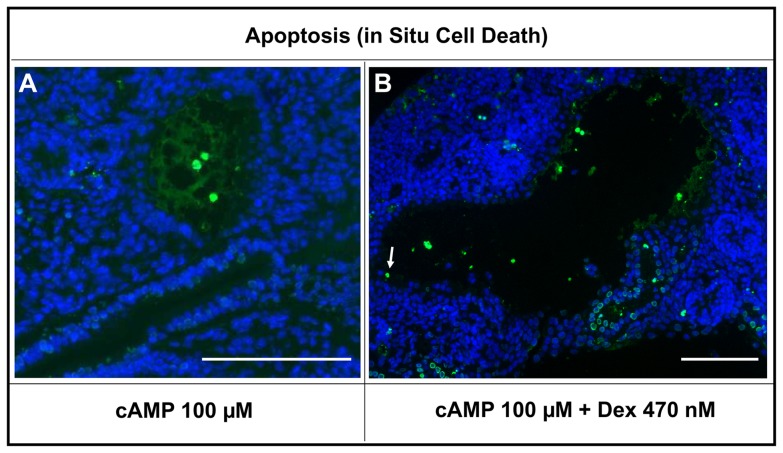
Apoptosis as assessed by TUNEL labeling. Histology images of day six cultures probed to detect apoptotic nuclei (green) with all nuclei counterstained blue. The left hand frame (**A**) is an image of an organ exposed to 100 µM 8-Br-cAMP-alone, and the right hand image (**B**) is of an organ exposed to this chemical plus 470 nM of dexamethasone. Note apoptotic nuclei in cyst lumens together with rare apoptotic nuclei (arrow in B) in epithelia lining cysts.

Explants were maintained in culture for six days, and further analyses were undertaken (Figures 7, 8, and 9). In whole-mounts, cystic structures were observed in all conditions apart from vehicle or 4.7 nM dexamethasone alone. There was no significant difference in percentage areas occupied by cysts between embryonic kidneys exposed to 47 nM or 470 nM dexamethasone alone or to 100 µM 8-Br-cAMP alone (respective median cystic areas being 5%, 5% and 2%). Notably, cultures treated with 100 µM 8-Br-cAMP plus the lowest concentration (4.7 nM) of dexamethasone showed a significantly increased cystic area (median 12%) compared with those exposed to 100 µM 8-Br-cAMP alone. When 47 or 470 nM dexamethasone was co-administered with 100 µM 8-Br-cAMP, there occurred further, major enhancements of cystogenesis (respective median cystic areas being 38% and 40%). The spectrum of severity of cystogenesis can be appreciated in both whole mount images (Figure 7B) and in low power microphotographs (Figure 9A–D). Epithelia lining cysts in organs exposed to 470 nM dexamethasone alone, 100 µM 8-Br-cAMP alone, or a combination of the two, generally reacted with antibodies to megalin (Figure 9E–H) and aquaporin-1 (data not shown), but never to uromodulin antibody (Figure 9I–L) and rarely to calbindin-28 antibody (Figure 9M–P). The *Dolichos biflorus* lectin (Figure 9Q–T) prominently labeled non-dilated tubules between cysts; in addition, the great majority of cysts did not bind the lectin. Proliferating nuclei were rarely detected in cyst epithelia (data not shown). Apoptotic nuclei were noted in cyst lumens (Figure 10) but co-administration of the general caspase inhibitor Z-VAD-FMK (25 µM or 100 µM) [27] with either 8-Br-cAMP and/or dexamethasone did not abolish cyst formation (data not shown).

**Figure 11:**
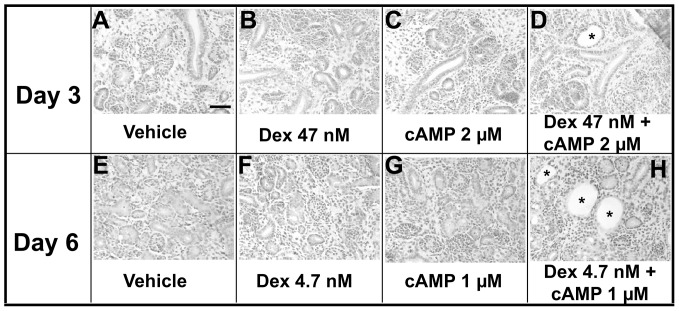
Histology of rudiments exposed to low concentration of cystogenic chemicals. **A–D.** After three days in culture, neither 47 nM dexamethasone (*Dex*) nor 2 µM Br-cAMP (*cAMP*) alone resulted in cyst formation; however, in combination, small cysts formed (asterisk in D). **E–H.** On day six of culture, neither 4.7 nM dexamethasone nor 1 µM Br-cAMP alone resulted in cyst formation; however, in combination, numerous cysts were noted (asterisks in H). Bar is 50 µm.

We used histology to determine the lowest concentrations of 8-Br-cAMP or dexamethasone which were cystogenic. On day three of culture (Figure 11A–D), neither 47 nM dexamethasone nor 2 µM 8-Br-cAMP alone resulted in cyst formation; however, in combination, these chemicals generated small cysts. On day six of culture, (Figure 11E–H), neither 4.7 nM dexamethasone nor 1 µM 8-Br-cAMP alone resulted in cyst formation; however, in combination, they were markedly cystogenic.

**Figure 12:**
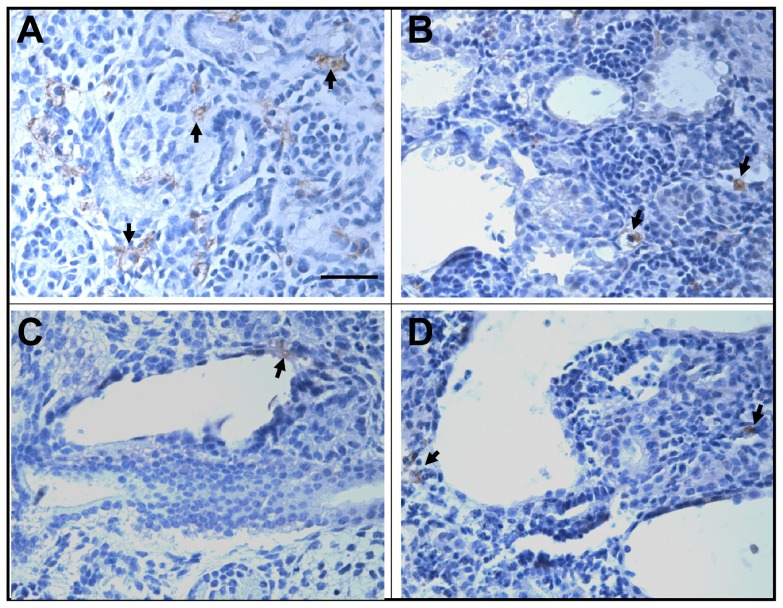
F4/80 immunostaining of macrophages in rudiments at day 6 of culture. (**A**) Vehicle-only exposed organ. (**B**) Dexamethasone-only exposed organ. (**C**) 8-Br-cAMP-only exposed organ. (**D**) Organ exposed to both cystogens. Immunoreactive cells, presumed macrophages, are brown and some are indicted by arrows. Note that F4/80 macrophages were detected in all four conditions. In cystic explants, their numbers did not appear increased and, moreover, they were often located distant from cyst epithelia. Bar is 50 µm.

As amplified in the *Discussion*, below, macrophages have been implicated in PKD models. Accordingly, explant tissue sections were immunoprobed with F4/80 antibody. Macrophages could be detected at day 3 and 6 of culture in all four conditions. In vehicle-only treated explants (Figure 12A), they were detected between nascent tubules. In explants exposed to dexamethasone alone (Figure 12B), 8-Br-cAMP alone (Figure 12C) or both these cystogens (Figure 12D), F4/80 macrophages were generally located away from cysts themselves and, although not formally quantified, they did not appear to be increased in number versus vehicle-only exposed organs. Other explant sections were probed with two different antibodies reactive to the mannose receptor, a M2 marker. However, we failed to detect positive immunostaining in any condition.

## Discussion

Exposure to 14 µM hydrocortisone generates cysts in metanephric organ culture [20], [21]. Hydrocortisone, or cortisol, is secreted into the circulation by the adrenal gland, and it has equipotent glucocorticoid and mineralocorticoid activities [36]. Glucocorticoid and mineralocorticoid receptor transcripts are both expressed in metanephroi, with the proteins immunodetected in tubules [21]. The cystogenic effect of hydrocortisone in metanephric culture has, however, been attributed to its glucocorticoid activity [21]. So, in this study, dexamethasone, a glucocorticoid-specific synthetic steroid, was used rather than hydrocortisone. As assessed by imaging whole mounts and histology, at day three of culture, only the highest concentration of the steroid caused mild tubule dilatation. 47 or 470 nM dexamethasone alone each resulted in cyst formation at six days of culture; exposure to 4.7 nM dexamethasone, however, was not cystogenic even at six days. As assessed by segment-specific markers, dexamethasone generally induced cysts in the proximal part of developing nephrons, a finding concurring with Avner *et al* (20) who microdissected hydrocortisone-exposed metanephroi. Chan *et al* [21] reported that exposure of cultured metanephroi to hydrocortisone resulted in altered levels of numerous transcripts coding for molecules involved in nephrogenesis and tubule maturation. In this context, chemical inhibition of either Na^+^K^+^ATPase activity [37] or growth factor signaling [21] reduces cystogenesis.

In contrast to dexamethasone-induced cystogenesis, 100 µM 8-Br-cAMP alone was cystogenic at both three and six days. Indeed, Magenheimer *et al* [25] reported that 8-Br-cAMP began to elicit tubule expansion in the first day of exposure. In accord with this previous study [25], we concluded that 8-Br-cAMP induced cysts in proximal nephron segments. The mechanism of cAMP-induced cystogenesis in metanephric culture has been shown [25] to depend on protein kinase A-driven activation of the cystic fibrosis transmembrane conductance regulator chloride channel. *Pkd1* ^−/−^ explants are hypersensitive to cAMP-induced proximal tubule cystogenesis versus wild type organs [25]. Notably, early cysts forming in human ARPKD fetal kidneys can also involve proximal tubules [38], [39], and they have been reported in *Pkd1* mutant fetal mice *in vivo* [40]. When ADPKD [41] and ARPKD [39] kidneys age, their collecting ducts become cystic. Indeed, the prominence of collecting duct-derived cysts accords with the anti-cystic effects reported in animals with PKD following pharmacological downregulation of AVP V_2_ receptor signaling [22]–[24]. In the current study, we observed only rare dilatation of tubule segments beyond the proximal nephron after six days of culture with either 8-Br-cAMP or dexamethasone alone or the combined agents. Moreover, supplementing vehicle with [Arg8] vasopressin was not cystogenic. Collectively, these results suggest that the newly formed proximal nephron is particularly susceptible to chemically-induced cystogenesis. Given that both apoptosis [42], [43] and proliferation [28] have been implicated in PKD, we undertook a limited set of experiments in which explants exposed to 8-Br-cAMP and/or dexamethasone were additionally treated with pharmacological blockers of apoptosis or proliferation. In the former case, although we cannot exclude a minor effect, cystogenesis appeared unabated; in the latter case, no conclusions could be reached because the drug resulted in major disruption of nephrogenesis.

The most novel findings of the current study involve the observations made when 8-Br-cAMP and dexamethasone were combined. Applied together, the degree of cystogenesis was markedly enhanced. After three days of culture in media supplemented with both 100 µM 8-Br-cAMP and 47 nM dexamethasone, the proportion of organ area occupied by cysts increased by about 50% versus the nucleotide alone, with a further significant increase when 100 µM 8-Br-cAMP was supplemented with 470 nM dexamethasone. Notably, at this time point, neither concentration of dexamethasone alone was significantly cystogenic; thus this was a synergistic rather than an additive effect. After six days of culture, the outcomes were even more striking. At this time point, whereas 4.7 nM dexamethasone alone was not cystogenic, when added with 100 µM 8-Br-cAMP, it more than doubled the cystic area elicited by the nucleotide on its own. After six days of culture, although 47 or 470 nM of dexamethasone were only modestly cystogenic on their own, when either was applied with 100 µM 8-Br-cAMP, cysts were found to occupy around 40% of the organ area, again a strikingly-synergistic effect. We also found that exposure to forskolin alone generated cysts and this effect appeared more prominent when it was applied with dexamethasone. Forskolin is a chemical which activates adenylate cyclase and which thus could potentially raise endogenously-generated intracellular cAMP levels.

We considered that the synergistic effects of dexamethasone and 8-Br-cAMP might have been explained by the glucocorticoid increasing the numbers of nephrons, so that more would be available to become cystic. At three days of culture, the organs were simple enough to be able to count cysts on whole mounts and numbers of cyst-like structures per explant were not significantly different between organs fed 100 µM 8-Br-cAMP alone or this chemical supplemented with any of the three concentrations of dexamethasone. Furthermore, we undertook a stereology-based approach to count glomeruli in day three cultures and found that numbers were similar in organs fed 100 µM 8-Br-cAMP alone versus those fed 8-Br-cAMP and 470 nM of dexamethasone. Of note, compared with organs fed vehicle alone or dexamethasone alone, organs exposed to 8-Br-cAMP tended to have fewer glomeruli, although this did not reach significance. In future, it would be of interest to count glomerular numbers in whole PKD kidneys but we are unaware of any such studies published so far. *In vivo*, exposure of rat or ovine fetuses to dexamethasone causes reduced numbers of glomeruli when assessed postnatally [44] or at term [45], a longer time-frame that the one covered in our current *in vitro* study. It should be noted that, because the organs in the current study underwent increasing complexity, cystogenesis and associated tissue distortion between days three and six of culture, we were unable to accurately measure numbers of glomeruli at the latter time point.

When 8-Br-cAMP and dexamethasone were applied together, a subset of cyst profiles contained glomerular tufts, meaning that the proximal tubule dilatation extended into the Bowman's space. Glomerular tufts attached to cyst walls were most obvious at day three of culture whereas, on day six, further cyst enlargement rendered it difficult to define residual glomerular tufts. A predominantly “glomerulocystic” phenotype is well-recognised to occur in a variety of contexts including experimental obstruction of fetal urine flow [46], [47] and certain genetic diseases such as the oral-facial digital syndrome [48] and the renal-cysts and diabetes syndrome [49]. Of note, kidney histology from fetuses and children carrying ADPKD mutations can feature prominent glomerular cysts [50], [51], as do fetal mice with *Pkd1* mutation [40]. Perhaps developing glomeruli are especially susceptible to undergo cystogenesis, and exposure to glucocorticoids can exacerbate a tendency initiated by physical (e.g. obstruction) or genetic (e.g. PKD mutations) factors.

There is interesting evidence emerging that macrophages may, at least in certain circumstances, be implicated in either driving kidney cystogenesis and/or in the response to cystogenesis. For example, Karihaloo *et al* [52] reported that F4/80 macrophages were prominently immunolocalised in the perimeters of kidney cysts in postnatal *Pkd1* mutant mice; moreover, cystogenesis was ameliorated in mutant PKD mice in which macrophages had been chemically-depleted [52]. Furthermore, we previously reported that F4/80 macrophages are detectable in wild type mouse metanephric explants [53]. Accordingly, in the current paper, we probed explants with F4/80 antibody. Although such macrophages could be detected in all four conditions (i.e. vehicle, 8-Br-cAMP and/or dexamethasone), they were generally located away from cysts themselves. Drugs such as dexamethasone may influence alternative (M2) activation of macrophages [54], and this macrophage system has been implicated in the biology found, for example, in a mouse model of ARPKD [55]. We therefore probed explants with two different antibodies reactive to the mannose receptor, a M2 marker. However, we failed to detect positive immunostaining in any condition. Collectively, the data do not support the idea that macrophages play major roles in cystogenesis in the current model, although definitive proof for this contention would require additional studies with macrophage-depleted organs exposed to chemical cystogens.

What are the implications of the current study for understanding human kidney cystogenesis? First, one needs to consider whether the concentrations of glucocorticoid used in the current study have any relevance to humans. Given that the glucocorticoid potency of dexamethasone is around 30 times greater than that of cortisol [36], 470 nM dexamethasone, the highest concentration used in this study, is equivalent to 14 µM cortisol in terms of glucocorticoid activity. This cortisol concentration is likely to exceed the highest levels measured in serum of severely stressed human adults [56]. Therefore, we also exposed embryonic kidneys to lower dexamethasone concentrations (4.7 and 47 nM), respectively equivalent to 140 and 1400 nM cortisol, thus spanning the physiological to stressed range found in adult human serum. The embryonic day 13 mouse metanephros is anatomically equivalent to a human kidney at six weeks gestation [57], [58]. During the culture period used in the current study, glomeruli begin to be generated, so that the explants anatomically resemble human embryonic kidneys of around eight to nine weeks gestation. Circulating cortisol levels have been measured in the second half of human gestation, with concentrations of between approximately 100 to 900 nM reported [59], [60]. While circulating cortisol levels have not, to our knowledge, been measured in human embryos in the first third of gestation, the initiating metanephros is likely to also be exposed to maternal-derived steroids with glucocorticoid activity.

We suspect that fetal overexposure to glucocorticoids alone is insufficient to be cystogenic *in vivo* and we note that kidney cysts have not been reported in animals exposed to dexamethasone during gestation [44], [45]. Instead, we speculate that antenatal glucocorticoid overexposure may enhance cystogenesis when an individual (or animal) already has a cystogenic tendency. The results of our current *in vitro* study provide biochemical evidence for an interaction between genetic (i.e. cAMP overactivity in PKD) and environmental (i.e. glucocorticoid excess in developmental programming) cystogenic agents. The data can be interpreted as initial evidence which supports the hypothesis that an adverse maternal environment may exacerbate PKD cystogenesis.

Human epidemiological data support a developmental origins, or fetal programming, theory of coronary heart disease [61], a concept which has been extended to help explain an individual's propensity to essential hypertension, obesity and insulin resistance [62]. As alluded to in the *Introduction*, antenatal overexposure to glucocorticoids may mediate at least some aspects of fetal programming. In certain experimental models, programming is associated with a low birth weight and, in human populations, it is notable that low birth weight is associated with cardiovascular disease in adulthood [61]. With respect to programming and cystic kidney disease in humans, two epidemiological studies are of particular interest. In a report from Norway [63], it was found that low birth weight for gestational age and intrauterine growth restriction increased the risk for end-stage renal disease (ESRD) when all kidney diagnoses were considered. Moreover, this association held in the disease subgroup with congenital or cystic nephropathy. Even more pertinent to the current study, a Danish study [64] noted that “for every kilogram increase in birth weight, the age at onset of ESRD significantly increased by 1.7 years”.

Lastly, we note that individuals with inactivating mutations of *11β-HSD2*, the gene which encodes an enzyme which protects the fetus and the kidney from cortisol, are prone to forming small cysts within their kidneys [65], [66]. This has been attributed to effects of chronic hypokalaemia, itself due to “apparent mineralocorticoid excess”. In view of the current study, we suggest that these kidneys may additionally be conditioned to become cystic by the glucocorticoid-mediated actions of cortisol beginning in the prenatal period.
